# Nonstructural Protein of Severe Fever with Thrombocytopenia Syndrome Phlebovirus Inhibits TBK1 to Evade Interferon-Mediated Response

**DOI:** 10.4014/jmb.2008.08048

**Published:** 2020-12-31

**Authors:** Jae Kyung Lee, Ok Sarah Shin

**Affiliations:** BK21 Graduate program, Department of Biomedical Sciences, Korea University College of Medicine, Seoul 08308, Republic of Korea

**Keywords:** SFTSV NSs, inclusion bodies, TBK1, innate immune response

## Abstract

Severe fever with thrombocytopenia syndrome virus (SFTSV) is an emerging phlebovirus of the *Phenuiviridae* family that has been circulating in the following Asian countries: Vietnam, Myanmar, Taiwan, China, Japan, and South Korea. Despite the increasing infection rates and relatively high mortality rate, there is limited information available regarding SFTSV pathogenesis. In addition, there are currently no vaccines or effective antiviral treatments available. Previous reports have shown that SFTSV suppresses the host immune response and its nonstructural proteins (NSs) function as an antagonist of type I interferon (IFN), whose induction is an essential part of the host defense system against viral infections. Given that SFTSV NSs suppress the innate immune response by inhibiting type I IFN, we investigated the mechanism utilized by SFTSV NSs to evade IFN-mediated response. Our co-immunoprecipitation data suggest the interactions between NSs and retinoic acid inducible gene-I (RIG-I) or TANK binding kinase 1 (TBK1). Furthermore, confocal analysis indicates the ability of NSs to sequester RIG-I and related downstream molecules in the cytoplasmic structures called inclusion bodies (IBs). NSs are also capable of inhibiting TBK1-interferon regulatory factor 3 (IRF3) interaction, and therefore prevent the phosphorylation and nuclear translocation of IRF3 for the induction of type I IFN. The ability of SFTSV NSs to interact with and sequester TBK1 and IRF3 in IBs demonstrate an effective yet unique method utilized by SFTSV to evade and suppress host immunity.

## Introduction

Severe fever with thrombocytopenia syndrome virus (SFTSV) is a tick-borne phlebovirus of the *Phenuiviridae* family that has been emerging in the following parts of Asia: Vietnam, Myanmar, Taiwan, China, Japan, and Korea [1-5]. The virus is transmitted by *Haemaphysalis longicornis*. *Rhipicephalus microplus*, *Amblyomma testudinarium*, and *Ixodes nipponensis* ticks [6-8]. *H. longicornis*, in particular, is the predominant tick species that bite humans, livestock, and poultry in regions where SFTSV is endemic, and plays an important role as a SFTSV vector [9]. Because ticks feed on animals, SFTSV can be maintained and transmitted to humans through both domestic and wild animals, among which sheep, dogs, and pigs are the major reservoirs [10, 11]. Human-to-human transmission is also a possible route for the spread of SFTSV [12]. Clinical manifestations following SFTSV infection can range from asymptomatic or mild to severe, and include high fever, thrombocytopenia, leukocytopenia, multi-organ dysfunction, and disseminated intravascular coagulation (DIC); multi-organ failure and DIC are characteristic symptoms of severe cases that result in high mortality rates and highlight the high viral replication rates of phleboviruses [11, 13, 14]. Similar to other members of the *Phenuiviridae* family, the SFTSV genome is comprised of three negative-sense, single-stranded RNA segments (small, medium, and large) that encode nucleoprotein and nonstructural proteins (NSs), glycoproteins, and RNA-dependent RNA polymerase. Despite the increasing prevalence and high mortality rate, ranging from 2-30%, SFTSV pathogenesis is not well described and there are currently no vaccines or antiviral treatments available for SFTSV infections [15].

The host innate immune response is important in controlling RNA viral infections, and virus recognition by the host innate immunity results in the activation of downstream signaling that leads to the activation of type I IFNs, IFN-α and IFN-β, which modulate the outcome of the viral infection [16]. RIG-I-like receptors (RLRs) constitute a major class of pattern recognition receptors (PRRs) that activate type I IFNs upon recognition of viral RNA containing a 5’-triphosphate modification [17]. This particular requirement of recognition allows the host immune system to differentiate between self and foreign RNA. Viral recognition by RLRs can lead to interaction with mitochondrial antiviral signaling protein (MAVS) to induce TBK1-dependent activation of transcription factor, IRF3, which upon activation, is translocated to the nucleus for transcriptional activation of IFNs [18].

In order to promote viral replication and persistence, viruses have evolved strategies to combat host antiviral response. SFTSV NSs encoded by the small (S) segment, in particular, have previously been shown to inhibit IFN-mediated JAK STAT pathways [19-22]. SFTSV has developed a mechanism to evade the host immune response through the interaction between NSs and type I IFN effector proteins, including TBK1 and MAVS, sequestering them inside SFTSV-induced cytoplasmic structures known as inclusion bodies (IBs) [19, 20, 22]. Furthermore, Moriyama *et al* showed that two conserved amino acids at positions 21 and 23 in the SFTSV and heartland virus (HRTV) NSs are essential for suppression of IRF3 phosphorylation and IFN-β mRNA expression following SFTSV infection [23].

In this study, we investigated the mechanisms utilized by SFTSV NSs to evade host type I IFN induction. Our findings confirm the interaction between NSs and RIG-I or TBK1, demonstrating the sequestration ability of NSs to withhold these molecules in the IBs. NSs also inhibit TBK1-IRF3 interaction, and therefore inhibit the phosphorylation and nuclear translocation of IRF3 for IFN activation. The ability of SFTSV NSs to interact with and sequester TBK1 and IRF3 in IBs demonstrate an effective yet unique method utilized by SFTSV to evade and suppress host immunity.

## Materials and Methods

### Cells

Human embryonic kidney HEK293T and HeLa cells were obtained from the American Type Culture Collection (ATCC; USA). HEK293T and HeLa cells were cultured in Dulbecco’s Modified Eagle’s Medium (Corning Mediatech, USA) supplemented with 10% fetal bovine serum (Corning Mediatech), 100 U/ml penicillin, and 100 μg/ml streptomycin.

### Immunoblot Analysis

HeLa cells (2 × 10^5^/well in a 6-well plate) were transfected with either a SFTSV NSs-MYC expressing plasmid (Sino biological, China) or an empty plasmid that served as control. At 24 h post-transfection, cells were subject to 2 h treatment with poly I:C (10 μg/ml) (Sigma-Aldrich, USA). After the incubation, cell lysis using radioimmuno-precipitation assay buffer (Sigma-Aldrich) supplemented with protease and phosphatase inhibitor cocktail (Roche, Switzerland) was performed and lysates were further prepared for immunoblot analysis. Polyvinylidene fluoride membrane activated with methanol was used for protein transfer from the sodium dodecyl sulfate polyacrylamide gel electrophoresis gel. Membrane was blocked with 2.5% skim milk in Tris-buffered saline buffer (0.2 M Tris, 1.36 M NaCl) with 0.1% Tween 20 and incubated with the appropriate primary antibodies (1:1000)(Cell Signaling Technologies, USA) overnight at 4°C, followed by incubation with secondary antibodies for 1 h at room temperature. Anti-tubulin antibody was used as the normalizing control (Abgent, USA). The membranes were washed extensively with Tris-buffered saline buffer with 0.1% Tween 20 between each step. Chemiluminescence was detected using Fusion Solo Imaging System (Vilber, France).

### Co-Immunoprecipitation

HEK293T cells were seeded in 6-well plates and transfected with the indicated plasmids. After 24-h incubation, cells were scraped and resuspended in Pierce IP Lysis Buffer (Thermo Fisher Scientific, USA) supplemented with protease inhibitor cocktail (Roche). Cell debris was removed by centrifugation (16,000 ×*g*, 4°C, 15 min). Antibodies were added and incubated with 50 μl protein A magnetic beads (SureBeads, Bio-Rad, USA) with rotation. The beads were washed extensively with PBS supplemented with 0.1% Tween 20. Beads probed with appropriate antibodies were incubated with 300 μg proteins 4°C with rotation. Proteins were analyzed by immunoblot analysis. Protein samples were probed with the following primary antibodies: anti-FLAG tag (1:2000; Fujifilm Wako Pure Chemical Corporation, Japan), anti-MYC tag (1:2000; Abcam, UK)

### Luciferase Reporter Assay

Luciferase reporter assay was performed as described previously [24, 25]. HEK293T cells were seeded at 5 × 10^4^ cells/well in 96-well plates and cultured overnight. The cells were transiently transfected with 50 ng of IFN-β, ISRE, IP-10, or IFN-λ1 luciferase reporter plasmid (IFN-λ1 reporter plasmids were kind gifts from Dr. Tomozumi Imamichi, Frederick National Laboratory for Cancer Research, Frederick, MD, USA), along with increasing dosage of SFTSV NSs in combination with an empty vector (EV), RIG-I, TBK1-specific plasmid, using Lipofectamine 2000 transfection reagent. EV plasmid was used to result in a constant total mass of DNA transfected. At 24 h post-infection, cells were lysed to measure luciferase activities using luciferase-reporter assay system (Promega, USA). The assay was performed in triplicate.

### Immunofluorescence Assay

HEK293T and HeLa cells were seeded on coverslips in 24-well plates. For CVB3 infection, CVB3 (H3 strain, Woodruff variant) [26] was quantified by plaque assay on HeLa cells as previously described [27]. At 24 h post-CVB3 infection or transfection with plasmids expressing SFTSV NSs, IRF3, TBK1, or RIG-I, cells were fixed with 4% paraformaldehyde, permeabilized with 0.1% Triton X-100, and blocked with 2.5% bovine serum albumin. Cells were stained with the appropriate antibodies to visualize the localization of SFTSV NSs and effectors of type I IFN signaling pathway. After the cells were labeled with the antibodies, 4,6-diamidino-2-phenylindole (DAPI) was used for counterstaining. Cells were washed between each step of the process with PBS. Coverslips were mounted on glass slides and imaged using Zeiss LSM700 (Carl Zeiss, Germany).

### Statistical Analysis

Student’s *t* tests were performed to compare individual treatments, and *p*-value < 0.05 was considered statistically significant.

## Results

### SFTSV NSs Is a Potent IFN Antiagonist

**Fig. 1:**
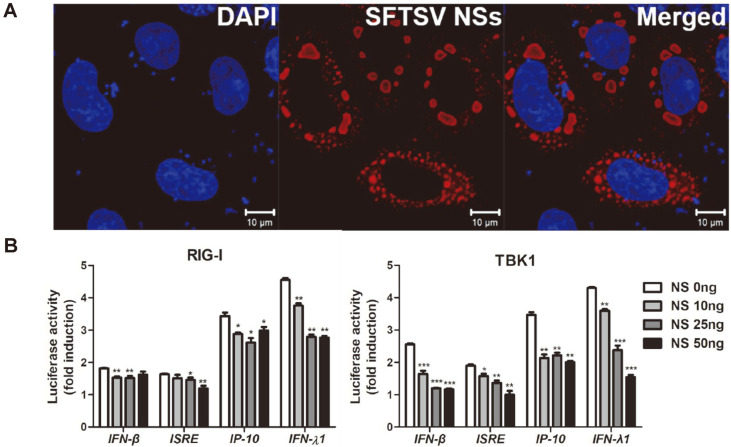
SFTSV NSs is a potent IFN antagonist. (**A**) HEK293T cells were transfected with SFTSV NSs-myc tagged plasmid. SFTSV NSs was detected by rabbit anti-myc tag antibody (red) and nucleus was stained with DAPI (blue). Scale bar, 10 μm. (**B**) Luciferase reporter assay of HEK293T cells transfected with IFN-β, ISRE, IP-10 and IFN-λ1 luciferase reporters together with RIG-I or TBK1 along with empty vector or increasing amount of SFTSV-NSs. Each error bar shows the means ± standard deviations of three independent experiments. *p* < 0.05; **p < 0.01; ***p < 0.001 versus empty vector-transfected cells.

We utilized a transient transfection system to determine if NSs expression alone can induce the formation of cytoplasmic IBs. When observed by confocal microscopy, expression of STSV NSs in HEK293T cells induces formation of IBs, which appear as round bodies dispersed throughout the cytoplasm (Fig. 1A). We next examined the effect of NSs on the host immune response in NSs-expressing HEK293T cells. Previous studies have confirmed the interaction of NSs and effectors involved in RLR signaling pathway including RIG-I and TBK1. Luciferase reporter gene assay was conducted to investigate if NSs block the activation of type I and III IFN (Fig. 1B). Co-transfection of SFTSV NSs and RIG-I or TBK1 encoding plasmids was carried out in HEK293T cells, in addition to IFN-β, ISRE, IP-10, or IFN-λ1 reporter plasmid. As shown, RIG-I and TBK1, when co-transfected with varying dosage of SFTSV NSs, inhibited the activation of IFN-β, ISRE, IP-10, and IFN-λ1 promoter activities. These findings indicate that SFTSV NSs expression can suppress both type I and III IFN promoter activation.

**Fig. 2:**
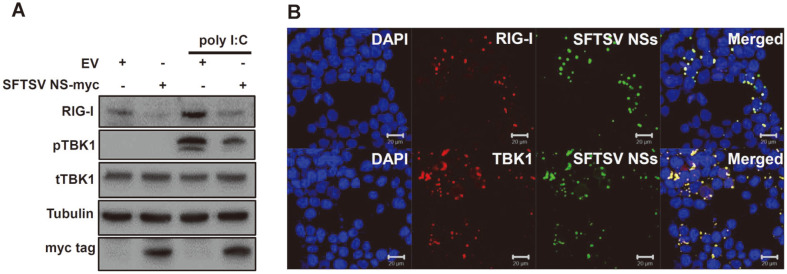
SFTSV NSs sequester RIG-I and TBK1 in NSs-induced inclusion bodies (IBs) and inhibit expression of RIG-I and downstream molecules in response to poly I:C treatment. (**A**) HeLa cells were transfected with SFTSV NSs-myc tagged or empty vector plasmid, and were subject to 2 h treatment with poly I:C (10 μg/ml) to mimic viral RNAinduced stimulation of the immune response. Indicated protein expression levels were analyzed by immunoblotting. Tubulin was used as a sample loading control. (**B**) HEK293T cells were co-transfected with SFTSV NSs-GFP tagged plasmid and RIG-IFLAG or TBK1-FLAG encoding plasmids. Co-localization of NSs was observed with RIG-I and TBK-1. Scale bars, 20 μm.

We also performed immunoblot analysis to study the effect of NSs on the expression of effectors of the innate immune response signaling pathway at the protein level (Fig. 2A). HeLa cells transfected with EV- or NSs-expressing plasmids were subject to poly I:C (10 μg/ml) treatment in order to mimic the viral RNA stimulation following infection. In NSs-transfected cells, expression levels of RIG-I and phophorylated TBK1 were found to be suppressed. The interactions between SFTSV NSs and RIG-I or TBK1 were also confirmed by confocal microscopy. NSs-induced formation of IBs was observed, in addition to the co-localization of IBs with RIG-I or TBK1 in the cytoplasm (Fig. 2B). These data demonstrate that NSs may directly or indirectly interact with RIG-I and TBK1.

### SFTSV NSs Interact with Effectors of Type I IFN Signaling Pathway

**Fig. 3:**
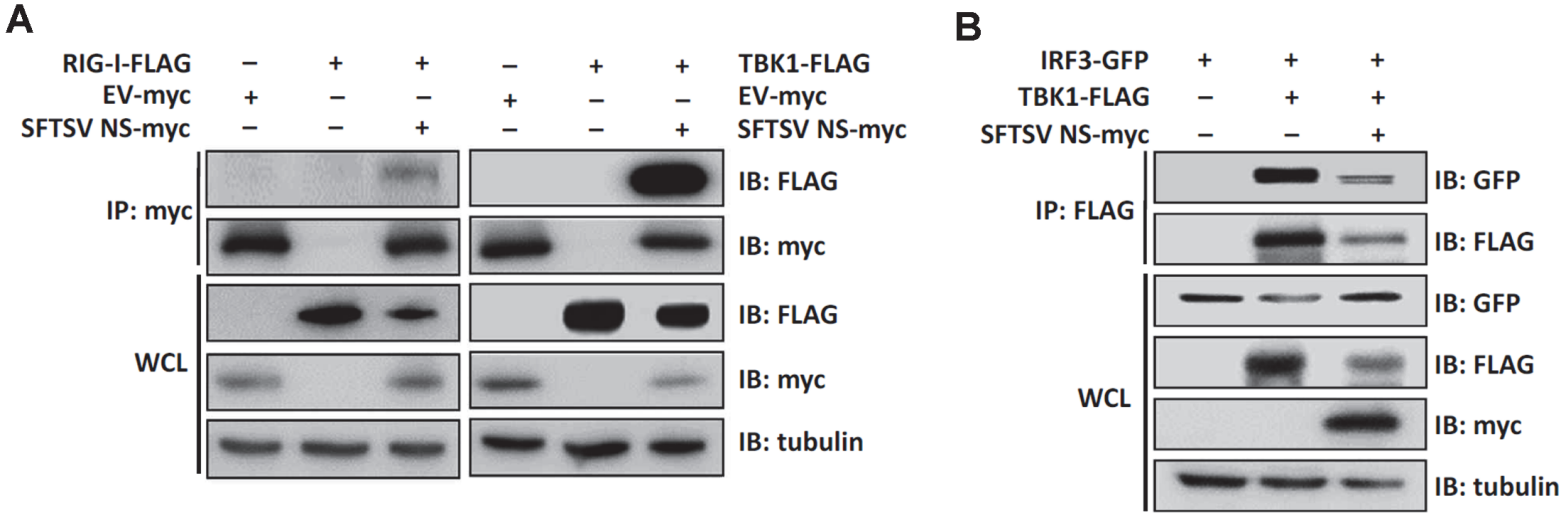
SFTSV NSs interfere with TBK1-induced IRF3 activation by inhibiting TBK1-IRF3 interaction. (**A**) HEK293T cells were transfected with plasmids encoding myc-tagged empty vector (EV) or SFTSV NSs and FLAG-tagged RIGI or TBK1. After 24 h post-transfection, cells were harvested and cell lysates were prepared for co-immunoprecipitation (IP) assay with rabbit anti-myc tag antibody. IP and whole cell lysates (WCL) were subjected to SDS-PAGE and immunoblot analysis with the indicated antibodies. The experiments were repeated three times. (**B**) HEK293T cells were transfected with plasmids encoding IRF3-GFP, TBK1-FLAG, and NSs-myc. Cells were harvested, lysed and immunoprecipitated with anti-FLAG tag antibody followed by SDS-PAGE and immunoblot analysis.

To investigate the mechanism utilized by NSs to suppress type I IFN response, we focused on discovering which cellular proteins associated with the innate immune response interact with NSs. HEK293T cells were transfected with SFTSV NSs and cell lysates were used for co-immunoprecipitation immunoblot analyses with antibodies targeting components of type I IFN signaling pathway (Fig. 3A). NSs coimmunoprecipitated with RIG-I and TBK1, and TBK1, in particular, is a crucial component of the IRF3 signaling pathway. Viral RNA recognition by RIG-I activates a signaling cascade that results in IRF3 activation, which occurs through phosphorylation, dimerization, and nuclear translocation of IRF3 [28]. IRF3-mediated induction of type I IFN is an essential aspect of the innate immune response.

### SFTSV NSs Suppresses IRF3 Activation Via Abrogating TBK1-IRF3 Interactions

**Fig. 4:**
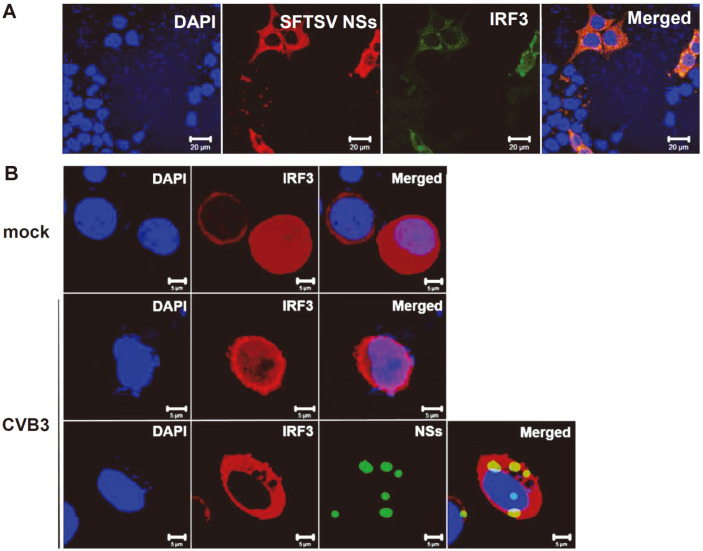
SFTSV NSs inhibit IRF3 nuclear translocation. (**A**) HeLa cells were co-transfected with plasmids encoding myc-tagged SFTSV NSs and FLAG-tagged IRF3. Confocal microscopy of cells stained with DAPI (blue), NSs (red), IRF3 (green). Scale bars, 20 μm. (**B**) Hela cells were stained with DAPI (blue), IRF3 (red), SFTSV NSs (green). IRF3 translocated to the nucleus in both mock- and CVB3-infected cells in the absence of NSs, whereas cells co-transfected with IRF3 and NSs remain in the cytoplasm following CVB3 infection. Cells were visualized using confocal microscopy at 24 hpi. Scale bars, 5 μm.

Since NSs sequester TBK1 in IBs and TBK1 acts as an important player of IRF3 activation, we examined if NSs also interfere with the interactions between TBK1 and IRF3. Co-immunoprecipitation assays revealed that NSs inhibit TBK1-IRF3 interactions (Fig. 3B). This finding indicates the ability of NSs to bind TBK1 with a stronger affinity and inhibit TBK1-induced activation of IRF3. The co-localization of NSs and IRF3 in the cytoplasm also suggest that NSs-induced IBs may also sequester IRF3 without directly interacting with IRF3 (Fig. 4A). IRF3 activation consists of TBK1-induced phosphorylation, dimerization, and nuclear translocation. Therefore, we also tested to see if NSs block the nuclear translocation of IRF3 in response to viral infection.

Coxsackievirus B3 (CVB3) is a well-known positive strand RNA virus of the Picornaviridae family that has been identified as a causative agent of myocarditis and pancreatic injury [29]. Similar to SFTSV, CVB3 RNA recognition by RIG-I and MDA5 leads to the activation of IRF3-mediated type I IFN response [27]. Nuclear translocation of IRF3 was confirmed in HeLa cells infected with CVB3, in comparison to mock-infected cells, in which IRF3 expression was localized in the cytoplasm (Fig. 4B). In cells co-transfected with NSs and IRF3, CVB3 infection did not result in the nuclear translocation of IRF3, as indicated by the co-localization of NSs and IRF3 in the cytoplasm. NSs expression alone was able to inhibit the nuclear translocation of IRF3 in response to a viral infection. Inhibition of IRF3 nuclear translocation in NSs-expressing, virus-infected cells further indicate the potential of NSs-induced IBs to sequester IRF3, as previously stated.

## Discussion

In this study, we investigated the role of SFTSV NSs in viral pathogenesis and host immune evasion. Despite the high mortality rate and severity of symptoms, including multiple organ failure, vaccines and proper treatment for SFTSV infection remain to be developed. Therefore, it is important to gain deeper understanding of the mechanisms utilized by SFTSV in evading the host innate immune response and identify which host proteins the virus interacts with during this evasion process.

NSs of *Phenuiviridae* family have been identified as the major virulence factor for phleboviruses once inside the host. Previous studies have reported the immunosuppressive roles of NSs, especially as IFN antagonists [19-22]. Similar to this finding, our data demonstrate that SFTSV NSs have an immunosuppressive role in the IFN pathway. Our results indicate that SFTSV NSs interact and co-localize with host signaling proteins, such as RIG-I and TBK1, findings that have also been confirmed in other studies, and suggest that SFTSV NSs inhibit the activation of IFN-induced host innate immune response through the RLR signaling pathway (Graphical highlights). Consequently, IRF3 is not phosphorylated, dimerized, or translocated into the nucleus, and IRF3-mediated activation of type I or III IFN is ultimately inhibited.

Previous reports have already highlighted the inhibitory roles of bunyavirus NSs, especially in terms of host immune evasion. Rift Valley fever virus (RVFV) is a well-known pathogen whose NSs have been identified as a virulence factor that inhibits IFN-β activation, and unlike SFTSV NSs, localizes in both the nucleus and cytoplasm and adopts a filamentous structure [30, 31]. Toscana virus (TOSV), Bunyamvera virus (BUNV), and La Crosse virus (LACV) express cytoplasmic NSs that inhibit type I IFN production by utilizing mechanisms such as inhibiting IRF3 nuclear translocation [32-36]. In addition to viral NSs being virulence factors that evade the host immune response and promote viral pathogenesis, NSs have also been associated with the formation of cytoplasmic structures known as IBs. Infections of cells by viruses, such as respiratory syncytial virus (RSV), can induce IB formation localized in the cytoplasm as part of viral survival strategy [37]. NSs of bunyaviruses can also form IBs that can function as site of sequestration of host factors associated with the immune response or as sites of viral replication [38]. In this study, we have confirmed the inhibitory role of SFTSV NSs in terms of suppressing the host innate immune response and the formation of IBs induced by NSs, even in the absence of infection. Our data have also shown that SFTSV NSs block IRF3 nuclear translocation, confirming that the virus is able to suppress the host innate immune response at the level of TBK1.

Our findings demonstrate that NSs-induced IBs are responsible for sequestering TBK1 and inhibiting IRF3-mediated activation of type I or III IFN response. SFTSV NSs disrupted the interaction between TBK1 and IRF3, and subsequently inhibited the phosphorylation, dimerization, and nuclear translocation of IRF3. TBK1 is also implicated in the activation of NF-κB, and p65, a subunit of NF-κB, is a potential target of TBK1 that is commonly associated with the inflammatory response. *Bunyaviridae* family of viruses has also been reported as inhibitors of NF-κB pathway [39]. Additionally, a recent study by Choi *et al* showed that SFTSV NSs targets the TPL2 signaling pathway to drive IL-10 cytokine production that facilitates immunosuppressive conditions to allow high viral replication, ultimately leading to viral pathogenesis [40].

In relation to SFTSV, Heartland virus (HRTV) is a phlebovirus whose NSs suppress IFN induction and promote viral pathogenesis. Ning *et al*. has shown that HRTV NSs target the innate immune response at the level of TBK1 and inhibit its interaction with IRF3 [41]. SFTSV pathogenesis has also been studied in small animal models, including mice and hamsters, and Liu *et al*. and Yamada *et al*. have demonstrated that type I IFN receptor knockout mice were highly susceptible to SFTSV infection [42, 43]. Findings from these animal models further confirm the importance of type I IFN signaling in terms of controlling viral replication and pathogenesis.

Aging is a major risk factor for SFTSV infection. A recent study by Park *et al*. suggests that gene expression profiles of SFTSV-infected young ferrets revealed strong interferon-mediated anti-viral signaling, whereas inflammatory immune responses were markedly upregulated and persisted in aged ferrets [44]. Given that multiple components of both innate and adaptive immune systems experience aging-related changes, it will be interesting to further study aging-associated impact on IFN modulation by SFTSV infection [45].

Future studies on the interactions between the host and SFTSV may reveal virus-induced modulation of the host immunity involving specific cellular targets and immunomodulatory factors beyond the RLR signaling pathway. SFTSV NSs was recently reported to mediate M2-skewed macrophage differentiation. Macrophages are important modulators of the immune system that are functional during both innate and adaptive immunity, and Zhang *et al*. suggests polarized differentiation towards M2 macrophages as a mechanism utilized by SFTSV to avoid clearance by host antiviral response [46]. Furthermore, SFTSV NSs-induced expression of microRNA-146b, which targets STAT1, was shown to inhibit M1 differentiation. These data indicate the diverse strategies utilized by SFTSV NSs to evade the host immune response and emphasize NSs as potential therapeutic targets for treating SFTSV infections.
